# kClust: fast and sensitive clustering of large protein sequence databases

**DOI:** 10.1186/1471-2105-14-248

**Published:** 2013-08-15

**Authors:** Maria Hauser, Christian E Mayer, Johannes Söding

**Affiliations:** 1 Gene Center and Center for Integrated Protein Science (CIPSM), Ludwig-Maximilians-Universität München, Feodor-Lynen-Str. 25, Munich 81377, Germany; 2 Department for Protein Evolution, Max Planck Institute for Developmental Biology, Spemannstr. 35, Tübingen 72076, Germany; 3 Present address: D-BSSE, ETH Zuerich, Mattenstr. 26, Basel 4058, Switzerland

## Abstract

**Background:**

Fueled by rapid progress in high-throughput sequencing, the size of public sequence databases doubles every two years. Searching the ever larger and more redundant databases is getting increasingly inefficient. Clustering can help to organize sequences into homologous and functionally similar groups and can improve the speed, sensitivity, and readability of homology searches. However, because the clustering time is quadratic in the number of sequences, standard sequence search methods are becoming impracticable.

**Results:**

Here we present a method to cluster large protein sequence databases such as UniProt within days down to 20%–30% maximum pairwise sequence identity. kClust owes its speed and sensitivity to an alignment-free prefilter that calculates the cumulative score of all similar 6-mers between pairs of sequences, and to a dynamic programming algorithm that operates on pairs of similar 4-mers. To increase sensitivity further, kClust can run in profile-sequence comparison mode, with profiles computed from the clusters of a previous kClust iteration. kClust is two to three orders of magnitude faster than clustering based on NCBI BLAST, and on multidomain sequences of 20%–30% maximum pairwise sequence identity it achieves comparable sensitivity and a lower false discovery rate. It also compares favorably to CD-HIT and UCLUST in terms of false discovery rate, sensitivity, and speed.

**Conclusions:**

kClust fills the need for a fast, sensitive, and accurate tool to cluster large protein sequence databases to below 30% sequence identity. kClust is freely available under GPL at http://toolkit.lmb.uni-muenchen.de/pub/kClust/.

## Background

In recent years, the amount of sequence data has been growing at an accelerating pace. While one would expect the denser sampling of sequence space to lead to better performance of sequence searches, the opposite seems to be true: The increase has led to stagnating or even negative returns, as measured by the ability to detect homologous sequences for structure or function predictions [1]. Removing redundant sequences through clustering can partly alleviate this problem: [2] and [3] showed that sequence searches through clustered databases, which contain one representative sequence per cluster, could improve the sensitivity of homology search methods. Furthermore, clustering reduces search times and can greatly improve the readability of search results [4]. Clustering is also often used in the analysis of metagenomics experiments, where potentially orthologous sequences are clustered together in order to define the inventory of biochemical reactions that are likely to be present in the metagenomic community [5-7].

An important motivation to develop kClust was our need for a database of profile hidden Markov models (HMMs) that contains all UniProt sequences, clustered down to 20%–30% sequence identity (uniprot20). Such a database is required by HHblits, the most sensitive protein sequence search method to date [8]. HHblits calculates a profile HMM from the query sequence and compares this query HMM with the uniprot20 database of HMMs. In subsequent iterations, sequences belonging to significantly similar database HMMs are added to the query multiple sequence alignment (MSA). Thanks to the additional information from homologous sequences in the MSAs, HMM-HMM comparison is more sensitive and accurate than profile-sequence comparison: Compared to PSI-BLAST [9], HHblits is faster, up to twice as sensitive, and produces alignments with several percent higher accuracy [8]. For HHblits it is critical that only a very low number of clusters are corrupted by non-homologous sequences, since these can cause high-scoring false-positive matches. The clustering sensitivity is also important because MSAs with higher sequence diversity and higher information content are better at finding remotely related sequences. Also, the lower number of clusters results in shorter search times.

Sequence clustering methods first need to compare the sequences in the input set with each other. Most methods use FASTA or BLAST [10,11] for this purpose. Some of these methods use the pairwise sequence similarities for hierarchical clustering [12-15]. Others use them to cluster sequences into orthologous or functionally similar groups [16-22]. FASTA and BLAST are two to three orders of magnitude faster than Smith-Waterman search [23] due to their fast, *k*-mer-based heuristic filters, yet they would require around 80 years of CPU time to cluster the UniProt database version with ∼35.5 million sequence from scratch. SIMAP [24] employs a 9-TeraFLOP distributed network of computers to regularly update their database of precomputed FASTA similarity scores for a large set of protein sequences. This database can be tapped to avoid the time-consuming sequence alignments [22], but it cannot be used for sequences not yet contained in SIMAP or when the clustering should depend on information other than sequence similarity scores.

CD-HIT [25-27] and UCLUST [28] are sequence clustering methods that, like kClust, do not rely on an external sequence search tool. All three methods aim at clustering large sequence databases much faster than what is possible using BLAST or FASTA. They start with zero clusters and pick one sequence after the other from the database. If the query is sufficiently similar to the representative sequence of a cluster, the query is added to that cluster. Otherwise it becomes the founding member and representative sequence for a new cluster. For the fast comparison of the query to the representative sequences of the clusters, these methods first prefilter the representative sequences using an alignment-free, *k*-mer-based sequence comparison. Sequences that pass the prefilter are aligned to the query using Smith-Waterman alignment. The clustering finishes when all sequences have been picked and assigned to a cluster.

For prefiltering, CD-HIT and UCLUST simply count the number of identical *k*-mers between sequences. Because this number drops quickly as the similarity of the compared sequences decreases, CD-HIT uses shorter *k*-mers for achieving higher sensitivity: (e. g. 5-mers for clustering thresholds down to 70% sequence identity, and 2-mers below 50%). The choice of *k* follows from the requirement for near-perfect sensitivity, between 95% and 99%. Reducing *k* comes at a considerable loss of speed, however, since decrementing *k* by one results in approximately 20 times more chance *k*-mers matches and a 20-fold longer run time. CD-HIT can therefore cluster large databases such as UniProt only to down to ∼ 50% sequence identity. UCLUST uses 5-mers at all clustering thresholds. This allows it to maintain a high speed even at low thresholds, at the cost of a loss of sensitivity. It ranks the representative sequences by the number of 5-mers they have in common with the query sequence and aligns them in this order until one of them is similar to the query sequence or until the highest-ranked eight clusters have been rejected. An apparent disadvantage of UCLUST is that, in order to increase sensitivity despite its word length of 5, it uses rather loose acceptance criteria. At clustering thresholds below 50%, this leads to a high fraction of corrupted clusters containing non-homologous sequences.

Both CD-HIT and UCLUST use banded dynamic programming to speed up the Smith-Waterman search. In addition, UCLUST extends the alignment around identical 5-mer matches in a way similar to BLAST [11].

kClust achieves high sensitivity by allowing matches between similar k-mers and ranking sequence pairs by the sum of similarity scores over all similar k-mer pairs.

We benchmark the performance of kClust and other tools to cluster large sequence databases well below 50% sequence identity. Our results show that kClust achieves false discovery rates similar to BLAST at much higher speeds, whereas at these low clustering thresholds CD-HIT and UCLUST manifest severe limitations in sensitivity, false discovery rate, and speed.

## Implementation

### Overview of kClust algorithm

kClust, like CD-HIT and UCLUST [26,28], uses the incremental, greedy clustering strategy of [29] (Additional file 1: Figure S1). First, all sequences are sorted by length. Starting with the longest one, the next sequence is picked from the database as query and is compared with the representative sequences representing the already created clusters. If the query fulfills kClust’s similarity criteria with one of the representative sequences, the query is added to that cluster, otherwise a new cluster is created for which the query becomes the representative sequence.

A fast, alignment-free prefilter reduces the number of costly computations of sequence alignments. Whereas CD-HIT and UCLUST count exact *k*-mer matches, kClust calculates the sum of similarity scores over all *similar* 6-mers. To increase speed, alignments are constructed with a novel algorithm, *k*-mer dynamic programming (kDP), which finds the local optimal alignment passing through pairs of similar 4-mers (C.E.M. and J.S., to be published). Furthermore, kClust employs spaced 6-mers and 4-mers that reduce the noise caused by the correlation between scores of neighboring *k*-mer matches [30].

Three similarity criteria are used to decide if a query is added to a cluster: (1) The sequence similarity score from the 4-mer-based dynamic programming algorithm is larger than a minimum BLOSUM62 score per column (default 1.12 half bits, which corresponds to a sequence identity of ∼30*%*, see Additional file 1: Figure S2), (2) the alignment achieves an E-value less than a defined threshold (default value 1E-3), and (3) the alignment covers at least 80% of the residues of the representative sequence. This criterion ensures that clusters contain sequences with nearly identical domain composition.

In a second iteration, profile-based kClust can also merge homologous clusters. It computes sequence profiles of the clusters generated in the previous step and uses these for scoring during the prefilter and alignment stages. This further improves the sensitivity without loss of speed.

### Prefiltering

**Figure 1:**
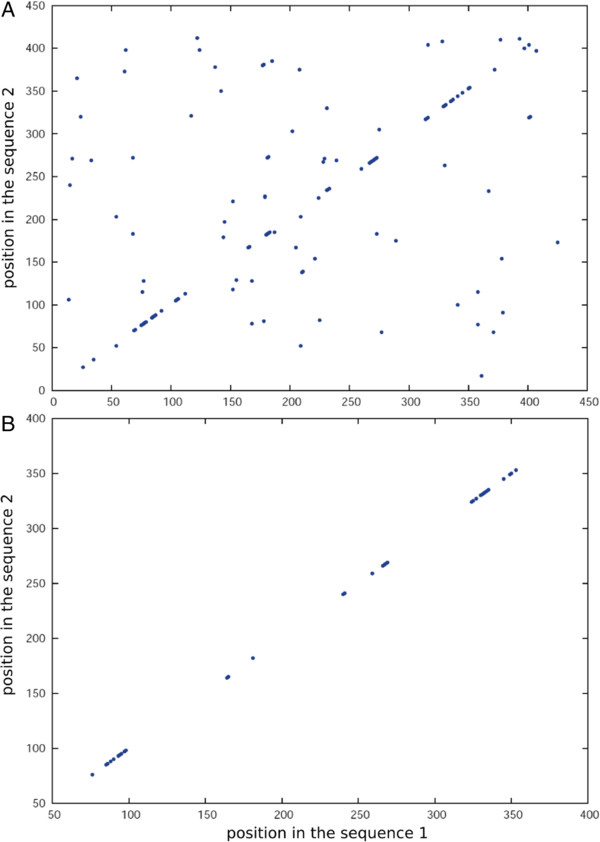
***k*-mer matches comparison.** Comparison of exact 3-mer matches **(A)** versus similar 6-mer matches with *r* = 100 **(B)** between two proteins with 45% sequence identity.

kClust’s prefilter sums up the substitution matrix scores of all 6-mers above a certain score threshold *S*_min_. (Default is 4.3 half-bits per column, or 12.9 bits, for a clustering threshold corresponding to roughly 30%, resulting in similar *k*-mer lists of an average length 100 per sequence position). As explained in detail in the supplementary discussion (Additional file 1), scoring *similar* *k*-mers confers a great advantage over counting identical *k*-mers. Briefly, by allowing non-identical matches a sufficient number of matches will occur even at low sequence similarities and for a word length as large as 6. On the other hand, the number of chance *k*-mer matches expected for unrelated sequences strongly decreases with increasing *k*, which in turn improves both the discrimination between true and false positives and the speed of the prefilter. This is illustrated in Figure 1, which shows the distribution of identical 3-mer matches and similar 6-mer matches for two proteins with a sequence identity of 45%.

**Figure 2:**
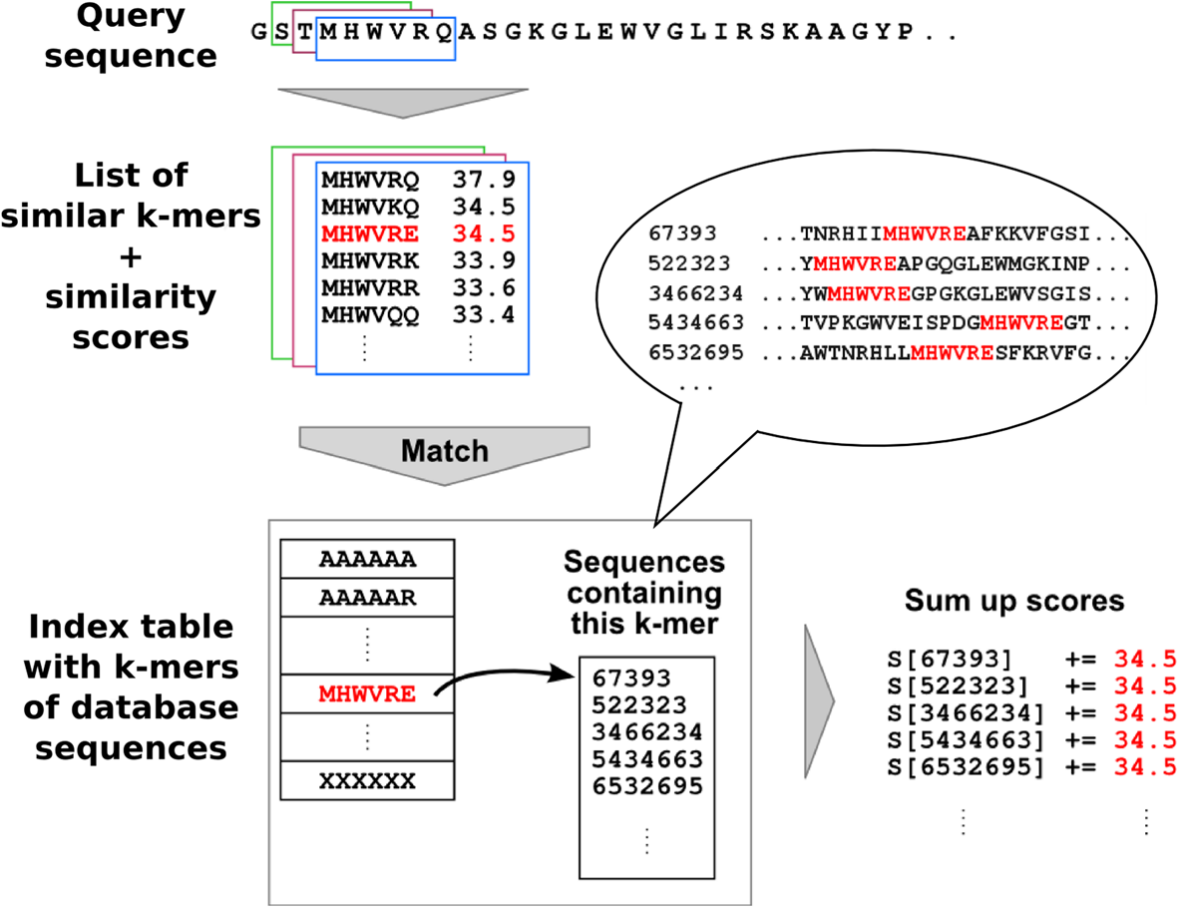
**Prefiltering step in kClust.** Prefiltering algorithm: For each *k*-mer in the query (*k* = 6), a list of similar *k*-mers and their BLOSUM62 similarity scores is generated (blue frame). For each such *k*-mer (red), a pointer to a list of representative sequences containing this *k*-mer is looked up in an array (index table). The score *S* of each sequence in that list is increased by the similarity score. After all *k*-mers in the query have been processed, array *S* contains for each representative sequence the sum of *k*-mer similarity scores.

The algorithm to calculate the sum of *k*-mer similarity scores is described in Figure 2. Sequences whose score is above a certain threshold are aligned to the query in the next step (kDP). The default prefilter score threshold is set to 0.55 half bits per query position, which results in a 98% sensitivity level (see Additional file 1: Figure S3). The array index in the index table at which the pointer for each *k*-mer (*x*_1_,…,*x*_*k*_) is stored is calculated as $\sum_{j=1}^{k}x_{j}|\Sigma {|}^{k-j}$, where |Σ| = 21 is the size of the amino acid alphabet.

#### Generating lists of similar *k*-mers

A branch-and-bound algorithm generates the list of all *k*-mers that have a BLOSUM62 score above a specified threshold *S*_*min*_. First, we calculate for each position *j* in the *k*-mer the minimum score *S*_min,*j*_ that is necessary to reach a total score above the threshold *S*_min_. This score is simply *S*_min_ minus the maximum score achievable for *k*-mer positions *j* + 1,…,*k* − 1:

(1) $$S_{\text{min},j}=S_{\text{min}}-\overset{k}{\underset{i=j+1}{\sum }}S_{\text{BLOSUM}62}(x_{i},x_{i})\;,$$

where *S*_BLOSUM62_(*x*,*y*) denote the BLOSUM62 substitution matrix scores. The list of similar *k*-mers is then generated iteratively position by position, by appending each of the 20 amino acids to the current *j*-mer and ending the branch if the score of the current *j*-mer is lower than the minimum required, *S*_min,*j*_. Using lists of amino acid dimers speeds up the process further by generating two amino acids instead of one at each step. Each dimer list is pre-sorted according to the score to the query dimer, so the branch can be skipped after the score of the *j*-mer falls below *S*_min,*j*_. Precomputed intermediate thresholds at each position and presorted dimer lists ensure that only *k*-mers with the overall score above the similarity threshold are generated. Since no *k*-mers except those with a score above the threshold are generated, this step has a time complexity of *O*(*r*).

#### Time complexity

The run time of the prefilter is the sum of two terms, the time to generate the lists of similar *k*-mers plus a term that is proportional to the number of *k*-mer matches between the compared sequences. The algorithm for generating the lists of similar *k*-mers has time complexity *O*(*r*), where *r* is the average length of the lists of similar *k*-mers. The second term stems from registering the *k*-mer matches with the representative sequences for each *k*-mer in the list. There are on average *N*_clu_*L*/20^6^ representative sequences containing a match to one query *k*-mer, where *N*_clu_ is the number of clusters (i.e. of representative sequences) produced in the clustering and *L* is their average length. The run time is therefore dominated by the second term, which has a time complexity

(2) $$O(N_{\text{db}}N_{\text{clu}}L^{2}p_{\text{match}})\;,$$

where *N*_db_ is the number of sequences in the database and *p*_match_ is the probability of a chance match above the similarity threshold between two *k*-mers. Note that before a new query sequence can be processed, the score array *S*[·] (see Figure 2) holding the prefiltering scores for the database sequences for this query has to be reset to 0. The naive resetting would result in a time complexity of $O(N_{\text{db}}^{2})$ which would be too time-consuming. For each query, we therefore start with an empty list to which we add all sequence indices whose score has been increased above zero, and we reset only the scores in that list.

### *k*-mer dynamic programming (kDP)

For the sequences that pass the prefiltering step, pairwise alignments are calculated using a fast heuristic, *k*-mer dynamic programming [31]. kDP records the similar 4-mer matches in the two sequences and determines the optimal alignment passing through the matched 4-mers. The optimal alignment is the one maximizing the sum of *k*-mer similarity scores minus gap penalties. In a second step, the full, residue-wise alignment with optimal BLOSUM62 score that passes through the 4-mers on the optimal kDP alignment is determined. Since kDP operates only on the similar 4-mer matches, the run time is proportional to number of matches, *p*_match_*L*^2^, where *L* is the length of the sequences. Hence, the run time can be reduced in principle by a factor of *p*_match_. In kClust, we chose the similarity threshold such that the lists of similar 4-mers have an average length of *r* = 200. Therefore, *p*_match_ ≈ *r* / 20^4^ ≈ 0.002 (see Additional file 1).

### Memory swapping

**Figure 3:**
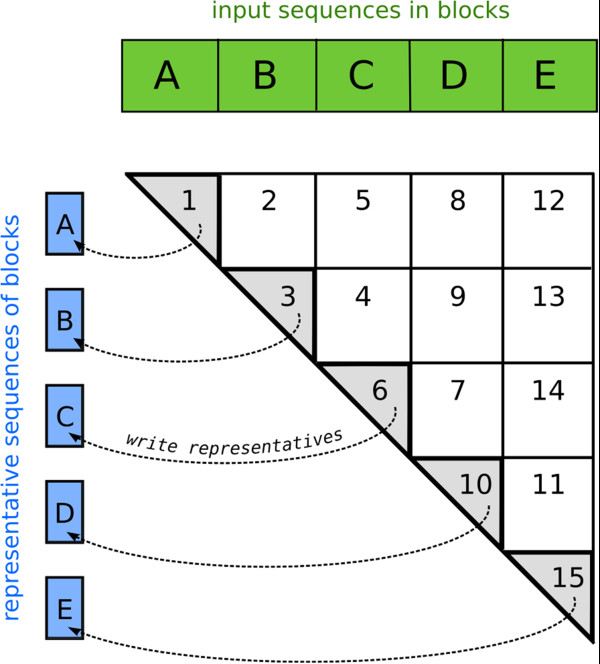
**Swapping.** Memory swapping procedure of kClust. Explanation see text.

The index table needs 21^6^ × 8*B* ≈ 650*MB* of main memory on a 64 bit system. The lists of indices of sequences containing the same *k*-mers take up a space of *N*_clu_*L* × 4*B*, or, assuming *N*_clu_ = 3*E*6 and *L* = 350, approximately 4*GB*. To allow kClust to run on computers with less memory, we have implemented a memory swapping procedure (s. Figure 3). The input sequences are divided into blocks (green). The first block is clustered with the usual clustering procedure, and a list of representatives is written to the hard disk (blue). Each following block is first compared to the representatives of the previous blocks (squares). In the last step, the remaining sequences are clustered producing a new list of representatives (triangles). With this procedure, kClust has only a part of the database in the main memory at each point of time.

### Iterative, profile-based clustering

**Figure 4:**
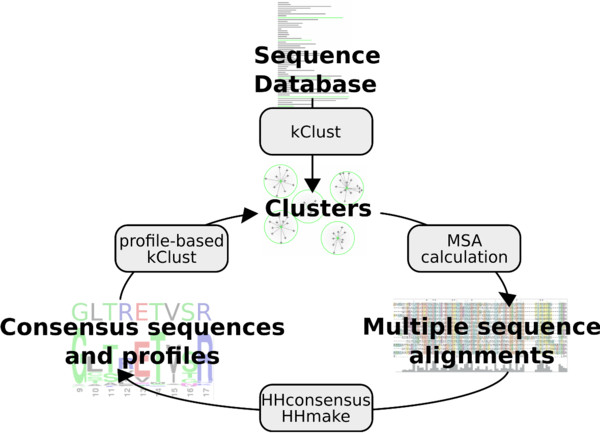
**Iterative kClust.** Overview over the iterative kClust method. First, kClust clusters the initial sequence database. Then, multiple sequence alignments are generated and profile and consensus sequences are computed for each cluster. Finally, profile-based kClust merges the clusters.

Iterative kClust is the extension of the basic kClust algorithm that allows to further merge clusters from the first clustering run (Figure 4). Iterative kClust compares clusters from the previous kClust run to each other instead of single sequences and merges clusters that are similar enough.

Multiple sequence alignments (MSAs) are generated for each cluster stemming from the previous iteration of kClust, and for each MSA a profile HMM and a consensus sequence are calculated with the hhmake and hhconsensus binaries of the open-source HH-suite software package [8]. The kClust algorithm proceeds in a similar way to the standard case, but instead of picking query sequences from the database, sequence profiles representing clusters are picked from the previously clustered database and compared to the clusters that have already been created in the present clustering iteration. As representative sequences of the clusters, the consensus sequences are used, which improves the sensitivity further [32]. The BLOSUM62 scores are simply replaced by the position-specific profile scores read from the profile HMM files. In the prefiltering, for instance, the lists and scores of the similar *k*-mers depend on the local 6-column window of the query profile. If no representative sequences similar to the query profile are found, the query is added as a new cluster to the database.

### Spaced seeds

**Figure 5:**
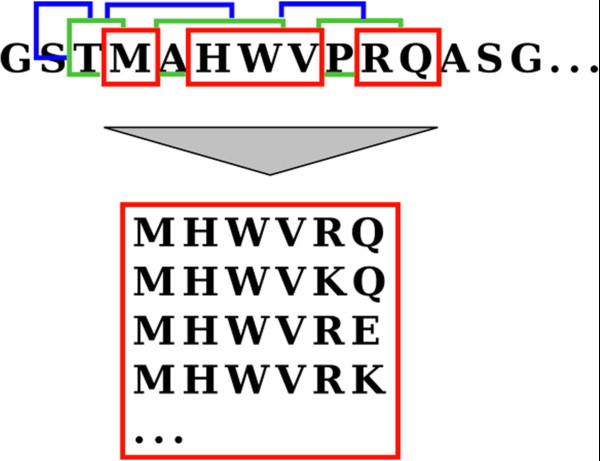
**Generation of spaced** ***k*-mers.** Spaced *k*-mers: The figure illustrates the generation of a list of spaced *k*-mers in the fast kClust prefilter algorithm (cf. Figure 2).

We use spaced instead of consecutive *k*-mers in the prefiltering and kDP steps. The generation of similar spaced *k*-mers for a sequence is illustrated in Figure 5. Spaced *k*-mers reduce the correlation of neighboring *k*-mer scores [30]. Therefore, *k*-mer matches are more evenly distributed and the probability of high scoring clusters of chance matches is reduced.

### Alignment generation and annotation of the clusters

The binary kClust_mkAln generates MSAs for each sequence cluster, using a user-specified multiple sequence alignment program. In addition, a cluster header with merged and redundancy-filtered names and annotations from the headers of the contained sequences is generated. The merging of UniProt and NCBI headers is supported.

## Results and discussion

### Benchmarked methods and parameters used

We compared kClust to three methods that are able to cluster large protein databases: CD-HIT, UCLUST, and BLAST-based clustering.

#### CD-HIT

We clustered the datasets with the newest, parallelized version of CD-HIT [27] on 16 cores down to a sequence identity of 40%, the lowest possible value. We used the -n 2 setting (*k*-mer word length) recommended for a clustering threshold of 40%. We set the minimum alignment coverage of the longer sequence to 80% with the -aL 0.8 option and the maximum available memory to 6000 MB with the -M 6000 option. We used parallel calculation on 16 cores of the computer by setting -T 0 (i. e. all available cores).

#### UCLUST

We ran UCLUST with the -id 0.3 and 0.4 options (“UCLUST 30” and “UCLUST 40”). Additionally, we used the -targetfract 0.8 setting for the minimum alignment coverage 0.8 of the longer sequence. We tried the -usersort option that should sort the input database by length, but this led to occasional segmentation faults. We therefore gave UCLUST a length-sorted database as input. As a side note, when not crashing, UCLUST produced worse results with the -usersort option than with a presorted database.

#### BLAST-based clustering

BLAST-based clustering was calculated using pdbfilter.pl from HH-suite (http://toolkit.lmb.uni-muenchen.de/HH-suite/), adapted to our input data. This simple procedure uses the same incremental greedy clustering strategy as CD-HIT, UCLUST, and kClust, but instead of comparing sequences using a prefilter and dynamic programming, parallel BLAST (option -a 8) was employed. The acceptance condition to add a query sequence to an existing cluster was an *E*-value below 1E-3 and an alignment that covers more than 80% of the representative sequence. No condition on the sequence identity is used, which corresponds to the “max sens” version of kClust.

#### kClust

Four parameter settings were tested that differed in the acceptance criteria for adding sequences to existing clusters. In all four cases, the maximum *E*-value was 1E-3 and the minimum alignment coverage was 80%, which are the default values. For “kClust 30” and “kClust 20”, kClust was run with a clustering threshold of 30% and 20%, respectively. “kClust sens” was run with maximum sensitivity, i.e., with a clustering threshold at 0%. For “kClust iter”, one profile-based kClust iteration was run after a single iteration with “kClust max sens” settings.

### Clustering Pfam

Pfam-A-seeds version 24.0 are the 500 361 sequences that define the manually curated 11 912 domain families of the Pfam data base [33]. Of these, 4011 Pfam families are further grouped into 458 clans – a collection of families that are thought to be evolutionarily related. There are therefore 8359 groups of homologous sequences in the Pfam-A seeds set.

A pair of sequences is considered to be incorrectly grouped together in one cluster if the sequences belong to different Pfam families and to different clans. We call such a cluster corrupted.

All benchmarked methods produce an order of magnitude more clusters than the possible 8359 groups (s. Table 1). The reason is that many homologous relationships are hard to detect because of the low sequence similarities within many Pfam seed alignments. All methods generate only few corrupted clusters, with the exception of CD-HIT. This is not surprising, since this dataset contains only single-domain proteins, yet the most false positive cluster assignments originate from mistaking local similarities as global.

**Table 1:** Pfam clustering results

	**#Clusters**	**#Corrupted clusters**	**%Corrupted clusters**	**#Wrong seqs per**	**Time**
				**corrupted cluster**	
BLAST	118 920	5	4.2e-3	2.4	66 h 2 m ^∗^
kClust iter	111 251	4	5.2e-3	4.0	28 m/1 h 47 m ^†^
kClust sens	153 721	8	5.2e-3	1.6	17 m
kClust 20	153 883	8	5.1e-3	1.6	16 m
kClust 30	169 533	5	2.9e-3	1.6	16 m
UCLUST 30	234 039	10	4.3e-3	1.0	2 m
UCLUST 40	244 568	8	3.2e-3	1.0	2 m
CD-HIT	170 750	4 086	2.39	1.39	5 h 26 m

Clustering results on PfamA-seed single-domain sequences. ^∗^The time is the sum of the run times of all 8 parallel threads. ^†^Includes the time for the calculation of multiple sequence alignments.

Two iterations kClust achieve the highest sensitivity, i.e., the lowest number of clusters. The second iteration of kClust reduces the number of clusters in the first iteration almost by 30% without increasing the number of corrupted clusters. BLAST produces slightly more clusters with a comparable error rate. However, there is a striking difference in the run times – two iterations kClust take 28 min while BLAST clustering takes 66 hours. UCLUST is the fastest method, but it clusters less effectively 1.5 times more clusters than kClust and twice more than BLAST. CD-HIT produces less clusters than UCLUST, but with the highest error rate and is slow.

### Clustering artificial two-domain sequences

Most eukaryotic sequences consist of two or more structural domains. Multi-domain sequence pose greater challenges to clustering algorithms because they tend to cluster sequences together if they are sufficiently similar within one domain, even though their other domains may be unrelated.

Currently, most protein sequences are only partly or not annotated with contained domains. To test the ability of the tools to correctly cluster multi-domain sequences, we need a dataset of multi-domain sequences where all the sequences are annotated over their entire length. It should contain a sufficient number of sequences with the same domain composition in order to form enough clusters and also enough sequences with only local similarities, so it would be not trivial. Currently, there is no real dataset that would meet these criteria.

To test the ability of the tools to correctly cluster multi-domain sequences, we generated an artificial dataset of 100 000 two-domain proteins. We used the SCOP25 database [34] (SCOP database filtered to maximum 25% pairwise similarity), downloaded from the ASTRAL server (http://astral.berkeley.edu/). For each seed sequence from SCOP, we searched for homologous sequence segments using two iterations of PSI-BLAST [9] in the non-redundant database from NCBI (*nr*) and filtered the results to 30% minimum sequence identity to the seed sequence from SCOP and to 70% maximum pairwise sequence identity, using the hhfilter binary from the HH-suite package [8], in order to achieve not too low and not too high sequence similarity within the homologous group. Additionally, the homologous sequences must cover at least 90% of the seed sequence.

Sequences that remained after filtering were merged into groups, in accordance to the SCOP family membership of the seed sequences, one group corresponding to one SCOP family. 1000 groups containing more than 100 sequences were drawn randomly, and from the list of these groups 1000 combinations of two different SCOP families were drawn (two-domain architectures). For each such two-domain architecture, 100 two-domain sequences were generated by concatenating two randomly drawn sequences from the corresponding family groups. Additionally, we rejected a sequence if the longer domain in the artificial sequence covered more than 80% of the overall sequence length.

Two sequences were considered to be grouped correctly in one cluster if they have the same domain architecture. Thus, the dataset could, in principle, be clustered into 1000 clusters of homologous sequences with the same domain architecture.

The results of the clustering are shown in Table 2. Almost a quarter of the clusters CD-HIT and UCLUST generate are corrupted, although usually only by few sequences. BLAST produces 2.6% corrupted clusters. The reason for these false positives is the tendency of BLAST to extend into the unrelated region the alignment of sequences having only one domain in common. A slightly positive score over a nonhomologous segment is sufficient to extend the alignment to such length that it satisfies the alignment coverage criterion of 80%. kClust produces very reliable clusters, with 0.1% or fewer corrupted. This is likely due to the conservative alignments produced by kDP, which rarely extend beyond the homologous regions.

**Table 2:** Multidomain proteins clustering results

	**#Clusters**	**#Corrupted clusters**	**%Corrupted clusters**	**#Wrong seq per**	**Time**
				**corrupted cluster**	
BLAST	6 977	186	2.66	1.5	3 h 21 m ^∗^
kClust iter	8 537	10	0.1	1.1	13 m/39 m ^†^
kClust sens	17 070	10	0.06	1.1	9 m
kClust 20	17 127	10	0.06	1.1	9 m
kClust 30	22 047	6	0.03	1.2	9 m
UCLUST 30	39 284	10132	25.79	1.7	30s
UCLUST 40	50 104	10512	20.98	1.4	40s
CD-HIT	29 163	6 234	21.37	1.89	43m

Clustering results on a set of 100 000 two-domain sequences constructed from 1000 domain architectures with 100 sequences each. ^∗^The time is the sum of the run times of all 8 parallel threads. ^†^Includes the time for the calculation of multiple sequence alignments.

BLAST is clearly the most sensitive method, generating only 6977 clusters. kClust produces 17 070 in the first iteration with the maximum sensitivity setting, and one additional iteration reduces this number to 8537 without increasing the number of false positives. kClust clustered the dataset in the first iteration in about 9 min, the second iteration takes additional 4 minutes (without the time needed to generate multiple alignments of the clusters and profiles that are necessary for the second iteration). BLAST-based clustering took about 3.5 hours. UCLUST is very fast, needing less than one minute, but is much less sensitive, producing clusters with only 2.5 sequences on average, compared to kClust 20 with 5.8 on average.

It is noteworthy that even BLAST produces significantly more than the 1000 clusters that are theore- tically possible. The reason is that many alignments between sequences within one architecture group do not fulfill the coverage criterion, because the longest sequence is often significantly longer than many of the other members of the group, and because BLAST, like all other local alignment methods, often generates alignments that do not cover the entire homologous region.

### Clustering the UniProt database

At the time of the benchmark calculation (June 2013), UniProt [35] contained 36 042 779 amino acid sequences with 11 786 916 970 residues and average sequence length of about 330 residues.

The results for the clustering of the UniProt database are shown in Table 3. Since clustering run time increases quadratically with the database size, only kClust and UCLUST are able to cluster UniProt down to 30% sequence identity within a few days. All-against-all single-threaded BLAST would need about 80 years for the clustering. kClust clusters UniProt in 13 days 12 hours and produces 5 663 658 clusters. UCLUST needs only 3 days 5 hours to cluster UniProt and produces 6 636 076 clusters. Both tools were run with 30% sequence identity threshold and otherwise default settings. kClust needs about 12 GB of main memory for the clustering, the memory consumption of UCLUST is about 26 GB.

**Table 3:** Clustering of the UniProt database

	**#Clusters**	**Time**
BLAST (estimated)	?	80 y
kClust 30	5 663 658	13 d 12 h
UCLUST 30	6 636 076	3 d 5 h

Results for the clustering of the UniProt database, containing 36 042 779 sequences. BLAST running time is estimated.

**Figure 6:**
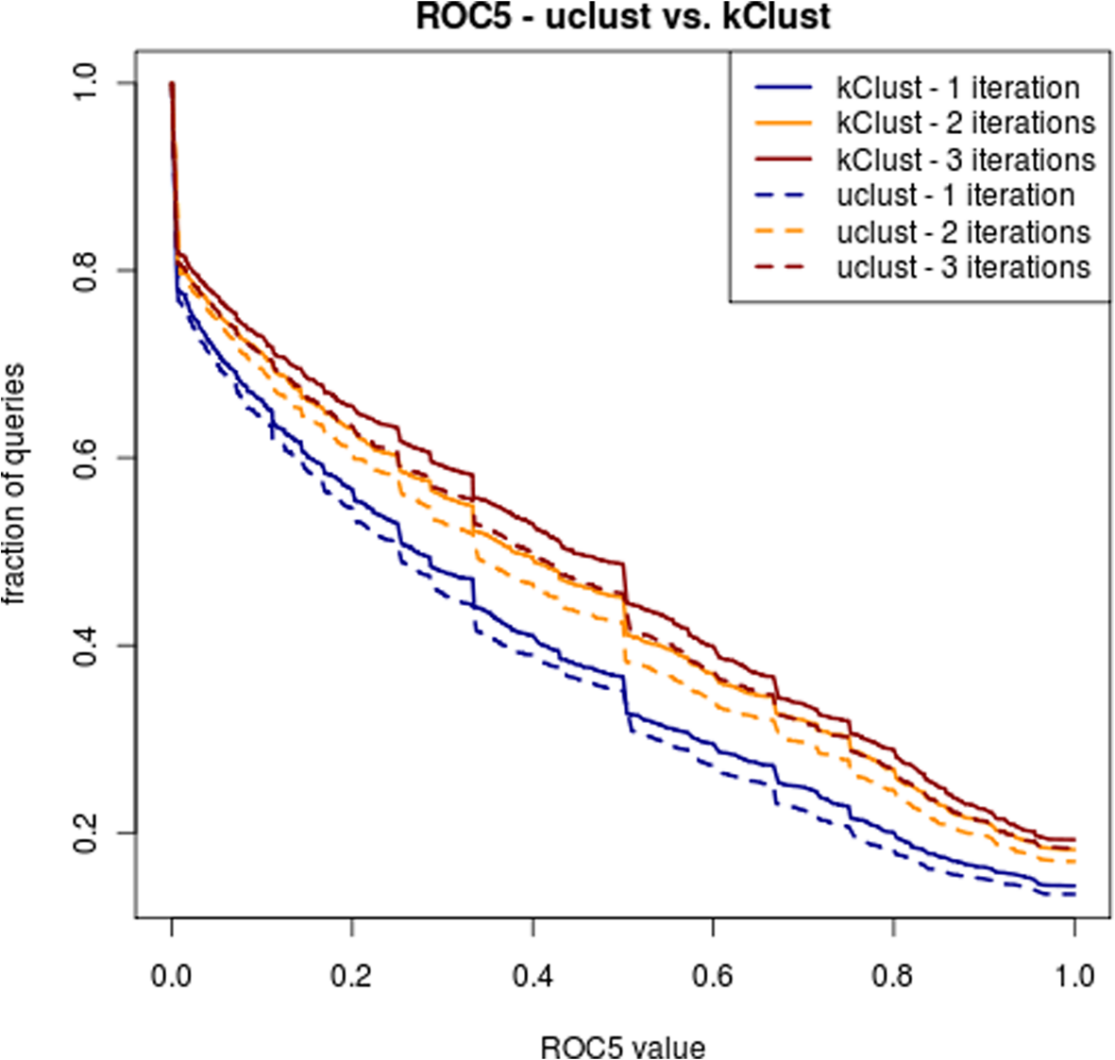
**Performance of HHblits on the clustered UniProtKB.** Fraction of queries with ROC5 value above the threshold on the x-axis, for one, two, and three HHblits iterations on the test set (5287 sequences from the SCOP 1.73 database). All but the last search iteration are performed against the UniProt. The last search iteration is done through a combined database containing the UniProt and the SCOP sequences. TPs are defined as pairs from the same SCOP folds, FPs as pairs from different folds, with the exception of Rossman folds and *β* propellers. The ROC5 value is the area under the ROC curve up to the 5th FP, normalized to yield a theoretical maximum of 1. The ROC5 plot is more robust to overfitting than the ROC curves.

We compared the quality of the clustering by checking the performance of HHblits on UniProt clustered with kClust or UCLUST, respectively (Figure 6). The performance is plotted after 1, 2 and 3 HHblits iterations. HHblits performs significantly better on UniProt clustered with kClust. 2 iterations of HHblits reach the same sensitivity on as 3 iterations on UniProtKB clustered with UCLUST.

### kClust running times on SwissProt

Running time of kClust depends heavily on the desired sequence identity threshold for the clustering. For lower thresholds, in addition to lowering the sequence identity threshold, kClust generates longer similar *k*-mer lists during the prefiltering and the alignment in order to increase sensitivity. As a consequence, the generation of the lists and index table matching takes longer.

**Figure 7:**
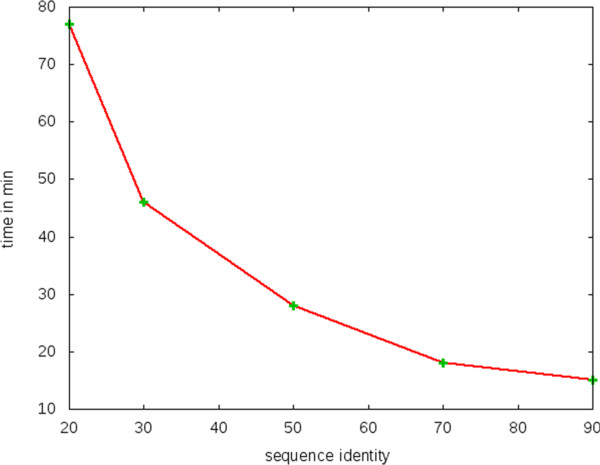
**kClust running time vs. clustering threshold.** kClust running times dependency on sequence identity threshold, calculated on SwissProt.

We performed a benchmark on SwissProt, a protein sequence database containing 540 261 sequences at the time of the benchmark calculation, and plotted kClust running times against the sequence identity threshold. The results are shown in Figure 7.

### Memory consumption of kClust and UCLUST

**Figure 8:**
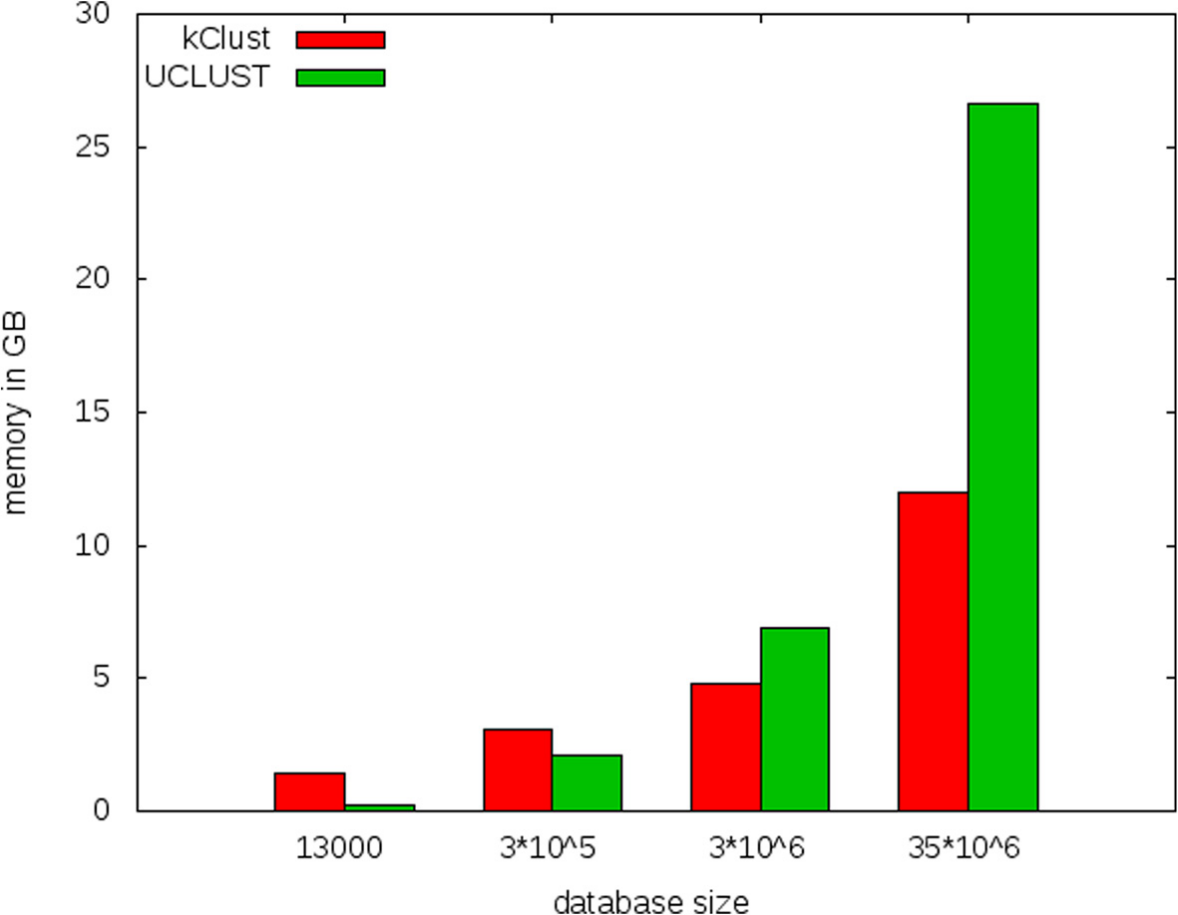
Memory consumption of kClust and UCLUST for different database sizes.

We performed a comparison of memory consumption of kClust and UCLUST for different database sizes. The results are shown in Figure 8. The different datasets are randomly chosen from the UniProt, the largest dataset is the whole UniProt.

## Conclusions

With the rapidly growing numbers of protein sequences from genome sequencing and metagenomics projects, methods that can cluster huge sequence sets in a reasonable time are in great need. Tools that are sensitive enough to cluster sequences together down to ∼30 % sequence identity will be particularly useful, since at that similarity protein domains usually still have the same or very similar molecular functions, in particular if their domain architecture is conserved [21,36]. Most existing sequence clustering methods rely on BLAST or FASTA for calculating the similarities between the sequences to be clustered, which makes them too slow for many clustering tasks ahead.

CD-HIT is fast and works well at high sequence identity clustering threshold, but it gets impracticably slow and inaccurate below 50% sequence identity. UCLUST is a very fast alternative which has, however, limited sensitivity as demonstrated in our study. kClust is to our knowledge the only method at present that fills the need for the fast clustering of large sequence databases or metagenomics sequence with a sensitivity not far from BLAST. kClust achieves this sensitivity with its prefilter that sums up the scores of similar 6-mers, its fast 4-mer-based alignment algorithm kDP, and its iterative, profile-based clustering strategy.

This is to our knowledge a novel approach to solving the problem of inexact, alignment-free comparison of sequences. A somewhat related approach that is popular in next-Generation sequencing data analysis is the “spaced seed” approach. In that approach, exemplified by PatternHunter [30] or SEED [37], one looks for exact matches between the compared sequences at nonconsecutive positions. Such a pattern of non-consecutive positions is called a spaced seed. Searches with several different such spaced seed patterns are then combined. For a given maximum number of mismatches, spaced seed sets can be constructed that guarantee an exact match for at least one spaced seed. This approach is very efficient as long as the number of allowed mismatches is low, say 4 out of 100. Our approach is applicable also in a range of 70 mismatches out of 100, far below what the spaced seed approach could handle due to the exploding number of spaced seeds required.

We are currently working on a faster and more flexible successor software which will be parallelized to run efficiently on multi-core architectures, which offers a more powerful clustering algorithm than the incremental, greedy clustering used in kClust, CD-HIT and UCLUST, and which will also allow to perform very fast parallelized sequence searches of a set of sequences against another set of sequences or sequence profiles. Importantly, it will allow updating of clustered databases with new sequences, thus obviating the need to recluster the entire sequence set from scratch.

## Competing interests

The authors declare that they have no competing interests.

## Authors’ contributions

CEM and JS designed the fast k-mer prefiltering, kDP, and kClust. CEM implemented the fast k-mer prefiltering, kDP, and noniterative kClust. MH designed and implemented the iterative kClust and the benchmarks. MH and JS wrote the manuscript. JS coordinated the project. All authors read and approved the final manuscript.

## Supplementary Material

Additional file 1 Supplementary material. Click here for file
