# Supplementary material for: kClust: fast and sensitive clustering of large protein sequence databases
Source: BMC Bioinformatics. 2013 Aug 15;14:248. doi: 10.1186/1471-2105-14-248 (PMC3843501; doi:10.1186/1471-2105-14-248)
Supplement: Additional file 1 — Supplementary material. [file 1471-2105-14-248-S1.pdf]

kClust: fast and sensitive clustering of large protein sequence databases – Supplementary Material

Maria Hauser [1,*], Christian E. Mayer [2,3,*] and Johannes Söding* [1]

[1]Gene Center and Center for Integrated Protein Science (CIPSM), Ludwig-Maximilians-Universität München, Feodor-Lynen-Str. 25, 81377 Munich, Germany. [2]Department for Protein Evolution, Max Planck Institute for Developmental Biology, Spemannstr. 35, 72076 Tübingen, Germany. [3]Present address: D-BSSE, ETH Zuerich, Mattenstr. 26, 4058 Basel, Switzerland.

*These authors contributed equally to the work.

Email: Maria Hauser - mhauser@genzentrum.lmu.de; Christian E. Mayer - christian.mayer@bsse.ethz.ch; Johannes Söding*- soeding@genzentrum.lmu.de;

*Corresponding author

Supplementary discussion: Similar versus identical k-mer matches

We show here that counting *similar* k-mer words for pairs of sequences can be much more sensitive than counting *identical* k-mers, because it allows us to keep the word length k large while still maintaining a high sensitivity for detecting similar pairs of sequences at low sequence identities. A higher word length in turn reduces much more the number of k-mers matching by chance than the number of k-mers matching as a result of the common ancestry of the two sequence segments. But kClust does not simply count similar k-mers, instead it sums up their BLOSUM62 similarity scores. This results in a further improvement in sensitivity, since k-mer pairs formed by chance will have lower scores on average than k-mer pairs that match due to their homology.

Consider first two homologous sequences with a sequence identity p_{seqid}, which we interpret as the probability that two homologous residues are identical. Assuming that the match probability of the k positions in a k-mer are approximately independent, the probability for two homologous k-mers to be identical is p_{seqid}^k. (In fact, since conserved positions are usually clustered in proteins, the true probability is actually larger than that, which will make the following estimates conservative.) For counting matches of similar k-mers, we demand that the score is larger than a certain threshold, such that for every k-mer there are on average r similar k-mers above this threshold (for example $r \approx 100$ in the 6-mer prefilter). The probability for two homologous k-mers to be similar is then approximately rp_{seqid}^k. To be able to detect the homology between two sequences, we need to count enough similar matching k-mers. If the sequences have a sequence identity p_{seqid} and their alignment has a length L, the number of expected matches must obey

$$rp_{\mathrm{seqid}}^k L \gg 1 \,. \tag{1}$$

The probability for two random k-mers to be identical is p_{ran}^k, where p_{ran} is the probability to observe two identical amino acids by chance. We estimate this probability using background amino acid probabilities $p_{\mathrm{bg}}(a)$: $p_{chance} = \sum_{a=1}^{20} p_{\mathrm{bg}}(a)^2 \approx 0.058$ Hence, the probability for two random k-mers to have a similarity above the threshold is rp_{ran}^k. A second necessary condition to be able to distinguish two homologous sequences of length L with sequence identity p_{seqid} from other, non-homologous sequences is that the homologous pair yield many more similar k-mer pairs than the number of chance matches $rp_{\mathrm{ran}}^k L^2$ in the pair of non-homologous sequences. Therefore, we must have

$$\left(\frac{p_{\mathrm{seqid}}}{p_{\mathrm{ran}}}\right)^k \gg L \,. \tag{2}$$

For $p_{\mathrm{seqid}} = 0.3$, the term under the power of k is $p_{\mathrm{seqid}}/p_{\mathrm{ran}} \approx 5$. Therefore, we gain a factor of five for each of position of the k-mer in the ratio of k-mer matches between homologous over non-homologous

sequences. We therefore should choose k as large as possible. In practice, k is limited by two considerations. First, the index table needs a memory of $21^k \times 8B$, which practically limits k to 6. Second, longer k necessitate exponentially larger list lengths r, and generating these lists of similar k-mers will dominate the total run time and limit the efficiency for $k > 6$.

For $k = 6$ we obtain $5^6 \approx 15\,000 \gg L$, showing that for sequences longer than 15 000 residues, the number of chance k-mer matches begins to outweigh the number of matches due to real homology. To estimate a suitable value r, note that eq. 1 tells us that we need to detect a sufficient number of matches even for short proteins. For a length $L = 50$, for example, the equation demands that $r \gg 1/(0.3^6 \times 100) \approx 40$. Hence $r = 100$ seems like a reasonable choice.

Figures

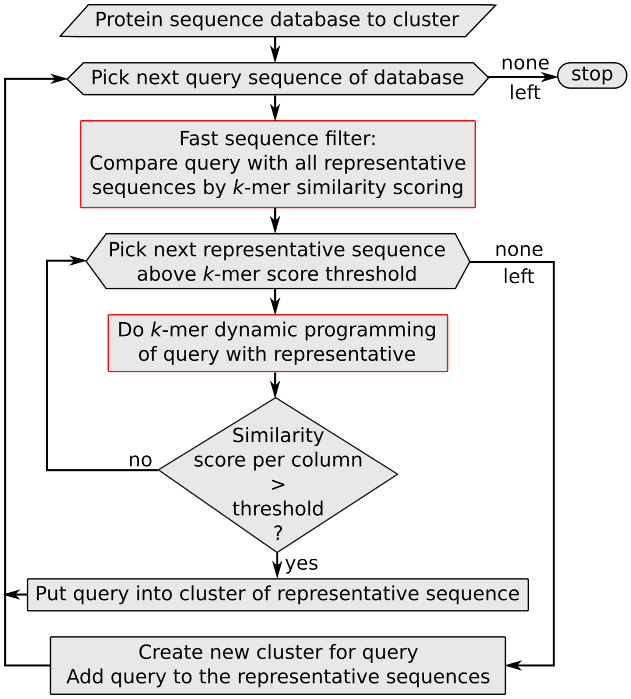

Figure 1: The upper red box represents the k-mer similarity scoring. Database sequences that passed the prefiltering step are compared to the query sequence with k-mer dynamic programming (lower red box).

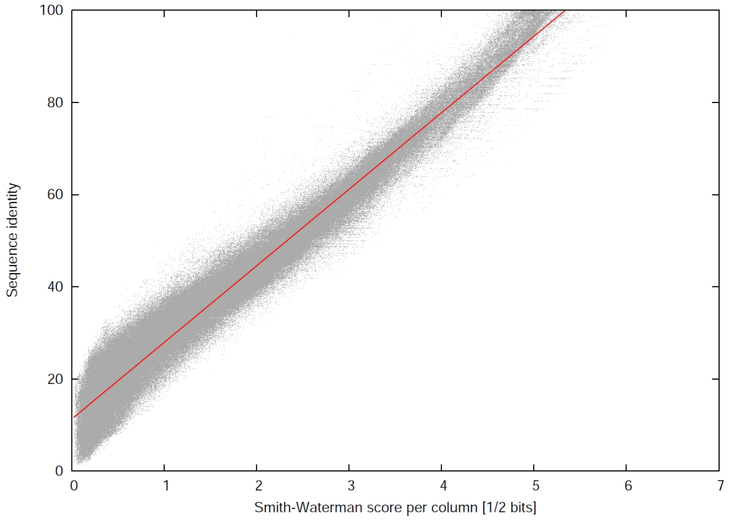

Figure 2: Correlation between the sequence identity and the BLOSOM62 alignment score per column. The sequence alignments are obtained by all-against-all Smith-Waterman alignment of sequences in SwissProt 51.0 which have pairwise BLAST E-values below 0.1. The linear regression, shown in red, is used to translate clustering threshold sequence identity values into score per column thresholds, which are used as one of the acceptance criteria to add sequences to clusters.

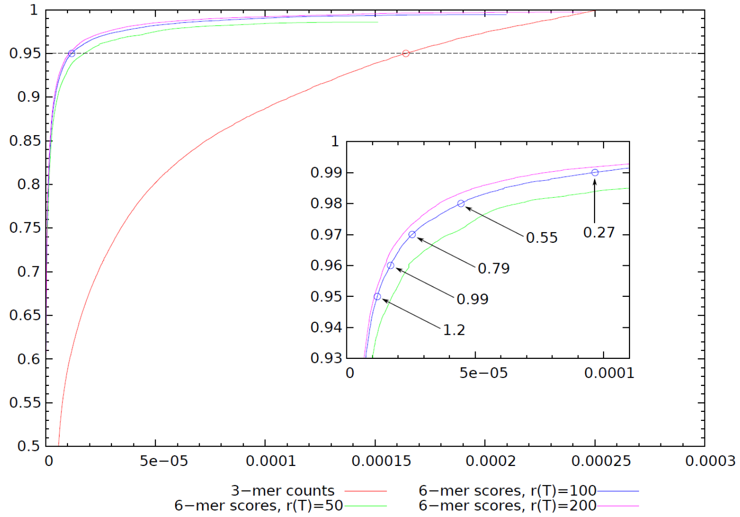

Figure 3: Receiver Operator Characteristic (ROC) curve for various prefiltering methods: Exact 3-mer matches and similar 6-mers for three different score thresholds corresponding to an average length r of the list of similar 6-mers of 50, 100, and 200.The inset shows a blow-up of the high sensitivity range above 0.93. The arrows point to various 6-mer thresholds in half-bits per residue. kClust default threshold is $T = 0.55$ half-bits, or $r(T) \approx 100$.
